# Osteopenia is associated with wasting in pancreatic adenocarcinoma and predicts survival after surgery

**DOI:** 10.1002/cam4.4416

**Published:** 2021-11-17

**Authors:** Miles E. Cameron, Patrick W. Underwood, Iverson E. Williams, Thomas J. George, Sarah M. Judge, Joshua F. Yarrow, Jose G. Trevino, Andrew R. Judge

**Affiliations:** ^1^ Department of Physical Therapy University of Florida Gainesville Florida USA; ^2^ Department of Surgery University of Florida Gainesville Florida USA; ^3^ MD‐PhD Training Program University of Florida Gainesville Florida USA; ^4^ Department of Medicine University of Florida Gainesville Florida USA; ^5^ Malcolm Randall VA Medical Center Gainesville Florida USA; ^6^ Department of Surgery Virginia Commonwealth University Richmond Virginia USA

**Keywords:** bone‐muscle interactions, cytokines, human association studies, prognostic markers, sarcopenia, tumor‐induced bone disease

## Abstract

Pancreatic ductal adenocarcinoma (PDAC) is among the deadliest of all common malignancies. Treatment is difficult and often complicated by the presence of cachexia. The clinical portrait of cachexia contributes to the poor prognosis experienced by PDAC patients and worsens therapeutic outcomes. We propose that low bone mineral density is a component of cachexia, which we explore herein through a retrospective review of all patients at our facility that underwent surgery for PDAC between 2011 and 2018 and compared to sex‐, age‐ and comorbidity‐matched control individuals. Data were abstracted from the medical record and pre‐operative computed tomography scans. Muscle mass and quality were measured at the L3 level and bone mineral density was measured as the radiation attenuation of the lumbar vertebral bodies. Patients with PDAC displayed typical signs of cachexia such as weight loss and radiologically appreciable deterioration of skeletal muscle. Critically, PDAC patients had significantly lower bone mineral density than controls, with 61.2% of PDAC patients categorized as osteopenic compared to 36.8% of controls. PDAC patients classified as osteopenic had significantly reduced survival (1.01 years) compared to patients without osteopenia (2.77 years). The presence of osteopenia was the strongest clinical predictor of 1‐ and 2‐year disease‐specific mortality, increasing the risk of death by 107% and 80%, respectively. Osteopenia serves as a test of 2‐year mortality with sensitivity of 76% and specificity of 58%. These data therefore identify impaired bone mineral density as a key component of cachexia and predictor of postoperative survival in patients with PDAC. The mechanisms that lead to bone wasting in tumor‐bearing hosts deserve further study.

## Graphical abstract

Bone density loss accompanies skeletal muscle atrophy in pancreatic cancer. Osteopenia is the single strongest predictor of 2‐year survival following curative surgery.

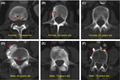

## 1 INTRODUCTION

Pancreatic ductal adenocarcinoma (PDAC) is among the deadliest of all common malignancies.^1^ Once considered a rare disease, PDAC is predicted to become the second most common cause of cancer death over the next decade.^2^ Significant barriers make the treatment of PDAC difficult including the high prevalence of cachexia in this cancer population. Cancer cachexia, a devastating syndrome characterized by profound skeletal muscle atrophy, with or without changes in adiposity, is present in 63% of patients at diagnosis and 80% of patients as the disease progresses.^3^, ^4^ Cachexia reduces the individual quality of life and tolerance to cancer treatment and is associated with adverse clinical outcomes and poor survival.^5^, ^6^, ^7^ Though there are multiple definitions of cachexia, most consider a combination of body weight loss together with reduced skeletal muscle mass and quality.^5^, ^8^ A multifaceted syndrome, cachexia accounts for the many metabolic disruptions, hormonal abnormalities, and immune system responses that collectively promote a negative energy balance, thereby facilitating skeletal muscle atrophy.^8^, ^9^, ^10^

Several studies have interrogated the relationships between muscle biology and bone density in normal aging and various neuromuscular diseases.^11^, ^12^, ^13^, ^14^ In brief, bone and muscle changes are believed to exist on a continuum driven by inflammation, common nutritional deficits, malnutrition, and reduced physical activity.^15^ The cross talk between bone and muscle is complex and represents an evolving field of study. Diverse endocrine signals emanate from skeletal and cardiac muscle, including cytokines such as interleukin‐6 (IL‐6), transforming growth factor‐beta (TGF‐β), and myostatin, that influence glucose metabolism, fatty acid oxidation, muscle growth, and osteoclast differentiation.^16^ Bone mineral density has not been thoroughly investigated in the presence of PDAC, though several factors that contribute to bone wasting have been thoroughly interrogated in PDAC‐associated muscle atrophy. Two studies, however, have considered osteopenia in the context of postoperative nutritional deficiencies and micro‐metastases.^17^, ^18^, ^19^ Several preclinical models have described reduced bone density and strength associating with muscle atrophy in the presence of cancer cachexia, although this has not been reported in the clinical literature.^20^, ^21^, ^22^, ^23^

The significance of osteopenia in PDAC and PDAC‐associated cachexia is largely unknown. Indeed, to our knowledge, there have been no clinical studies demonstrating the extent of bone loss in PDAC or cancer cachexia in general. To address this gap between the preclinical and clinical, we evaluated a comprehensive series of anthropometric measures in surgical PDAC patients and control individuals measured from routine, preoperative computed tomography (CT) scans.^7^, ^24^, ^25^, ^26^, ^27^ We hypothesize that patients with PDAC have lower bone mineral density than control individuals and that bone loss associates with cachexia. We further hypothesize that osteopenic patients have markedly worse long‐term survival than PDAC patients with healthy or age‐appropriate bone density.

## 2 METHODS

A prospectively maintained, IRB‐approved database was used to identify all consecutive patients who underwent resection for histologically proven PDAC between October 2011 and January 2018 at the University of Florida. Of the 162 patients identified, 152 had preoperative CT scans available and suitable for analysis. All patients had, at minimum, 2 years of postoperative follow‐up. Surgical complications and diverse oncologic outcomes were evaluable. Control subjects were randomly selected from age‐, sex‐, and Charlson comorbidity index‐matched individuals undergoing surgery for benign gallbladder pathology with appropriate abdominal CT scans.

We collected preoperative data including demographics (age, sex, race, and ethnicity), blood chemistries (complete blood count, comprehensive metabolic panel, and CA19‐9), PDAC signs and symptoms, neoadjuvant therapy regimen (if applicable), current and past medications, and comorbidities and basic body measurements such as height and weight. Postoperative pathologic data included tumor grade, tumor size, lymph nodes positive for PDAC, and surgical margin status. Surgical complications were recorded and graded according to the Clavien–Dindo index.^28^ Date of cancer recurrence and death were recorded if applicable.

### 2.1 CT Analysis

Preoperative CT scans were downloaded from the medical record, de‐identified, and saved as DICOM files. An axial image at the level of the third lumbar (L3) vertebra was accessed for each patient; the lower aspect of the L3 transverse processes was used to guide acquisition as cross‐sectional area at the L3 vertebra correlates with total body composition.^24^ Manual image segmentation was performed using sliceOmatic™ 5.0 (Tomovision, Magog, Quebec). Tissue thresholds were used that correspond to the radiodensities of skeletal muscle and adipose. Cross‐sectional skeletal muscle area (SMA, cm^2^) was calculated by the summative area between −29 and 150 Hounsfield Units (HU).^25^ Visceral, subcutaneous, and inter‐/intra‐muscular adipose cross‐sectional area were measured between −150 and −50 HU. SMA was normalized to height (m^2^) yielding a lumbar skeletal muscle index (SMI, cm^2^/m^2^). Individuals were classified as myopenic or not myopenic according to sex‐ and BMI‐dependent cutoffs of SMI as follows: SMI less than 43 cm^2^/m^2^ for men with a BMI less than 25 kg/m^2^, SMI less than 53 cm^2^/m^2^ for overweight or obese men, and SMI less than 41 cm^2^/m^2^ for women.^29^ Muscle radiation attenuation (MRA) was reported in HU. BMI‐dependent cutoffs of MRA were used to evaluate muscle health and degenerative, steatotic changes typical of atrophy, and cachexia of 41 HU for individuals with a BMI less than 25 kg/m^2^ and 33 HU for individuals with a BMI greater than 25 kg/m^2^.^29^ Inter‐/intra‐muscular adipose was reported as a percentage of total SMA. Total adipose (cm^2^/m^2^) was also reported as the summation of visceral and subcutaneous adipose cross‐sectional areas (cm^2^) normalized to height (m^2^). Individuals with PDAC were classified according to body weight loss in the preceding 6 months; among patients with greater than 5% loss, a subgroup was classified as cachectic based on prognostic variables including myopenia and MRA cutoff status and 8% body weight loss. All three criteria (significant body weight loss, low muscle mass, and low muscle quality) were used to stratify patients as cachectic in our analyses (see Table 1).

**TABLE 1:** Patient demographics and clinical variables of cachexia

	Variable	Control (*n* = 19)	Less than 5% body weight loss (*n* = 48)	More than 5% body weight loss (*n* = 104)	Cachexia^a^ (*n* = 38)	*p* value^b^	*p* value^c^	*p* value^d^
Demographics	Age (years)	64.2 (12.6)	68.3 (12.0)	67.9 (8.37)	70.0 (7.01)	0.2157	0.1029	0.0297
	Male, *n* (%)	11 (57.9)	25 (52.1)	62 (59.6)	22 (57.9)	0.7878	1.000	1.000
	Caucasian, *n* (%)	16 (84.2)	45 (93.8)	90 (86.5)	34 (89.5)	0.3406	0.7261	0.6754
Blood chemistries	Hemoglobin (g/dl)	13.3 (1.56)	12.6 (1.91)	12.4 (1.77)	12.1 (1.69)	0.1401	0.0386	0.0120
	Anemia, *n* (%)	4 (21.1)	24 (50.0)	52 (50.0)	20 (52.6)	0.0528	0.0242	0.0267
	Mean corpuscular volume (fl)	89.6 (3.27)	92.5 (6.59)	93.2 (5.10)	92.8 (5.73)	0.0746	0.0039	0.0310
	Platelet count (×10^9^/L)	202 (51.6)	259 (95.8)	261 (104)	253 (89.0)	0.0172	0.0167	0.0243
	Albumin (g/dl)	4.11 (0.597)	3.78 (0.555)	3.91 (0.570)	3.79 (0.459)	0.0364	0.1763	0.0325
	Creatinine (mg/dl)	0.863 (0.242)	0.855 (0.255)	0.881 (0.355)	0.864 (0.320)	0.9074	0.8386	0.9900
	Sodium (mmol/L)	139 (3.27)	139 (2.81)	136 (12.6)	135 (20.4)	0.9323	0.4829	0.4694
	Potassium (mmol/L)	4.08 (0.652)	3.91 (0.461)	4.03 (0.420)	4.07 (0.425)	0.2115	0.6556	0.9418
	Calcium (mmol/L)	9.07 (0.823)	9.20 (0.753)	9.35 (0.684)	9.32 (0.648)	0.5428	0.1190	0.2230
	AST (U/L)	37.8 (46.1)	70.2 (96.5)	61.7 (79.9)	79.4 (90.5)	0.1682	0.2098	0.0658
Bone history	Osteoporosis *n* (%)	2 (10.5)	2 (4.17)	7 (6.73)	3 (7.90)	0.3174	0.6281	1.000
	Vitamin D supplements, *n* (%)	2 (10.5)	3 (6.25)	12 (11.5)	2 (5.26)	0.6172	1.000	0.5942
	Tobacco use, *n* (%)	10 (52.6)	32 (66.7)	60 (57.7)	22 (57.9)	0.4011	0.8022	0.7809
Anthropometry	Body mass index (kg/m^2^)	29.4 (4.69)	27.0 (5.04)	26.5 (4.99)	25.7 (4.91)	0.0858	0.0202	0.0084
	Body mass index <20 kg/m^2^, *n* (%)	0 (0)	2 (4.17)	6 (5.77)	3 (7.90)	1.000	0.5888	0.5435
	L3 skeletal muscle index (cm^2^/m^2^)	46.1 (5.77)	43.8 (8.53)	43.9 (8.65)	38.7 (5.99)	0.2888	0.2742	<0.0001
	Myopenia *n* (%)	11 (57.9)	34 (70.8)	64 (61.5)	38 (100)	0.3894	0.8016	<0.0001
	L3 muscle radiation attenuation (HU)	34.5 (9.61)	32.0 (9.86)	30.7 (8.12)	25.9 (5.95)	0.3419	0.0717	<0.0001
	L3 muscle radiation attenuation below cutoff, *n* (%)	9 (47.4)	31 (64.6)	79 (76.0)	38 (100)	0.2701	0.0239	<0.0001
	L3 total adipose (cm^2^/m^2^)	144 (58.7)	131 (60.5)	124 (56.7)	123 (62.7)	0.4191	0.1650	0.2372
	Inter‐/intra‐muscular adipose (%)	6.78 (4.39)	6.78 (4.82)	6.78 (4.49)	8.53 (5.64)	0.9958	0.9964	0.2417
	Lumbar vertebral radiodensity (HU)	166 (44.0)	141 (36.6)	141 (46.2)	129 (5.64)	0.0197	0.0330	0.0025
	Radiologic osteopenia, *n* (%)	7 (36.8)	29 (60.4)	64 (61.5)	27 (71.1)	0.1059	0.0750	0.0214
	Signs of degeneration, *n* (%)	2 (10.5)	23 (47.9)	52 (50.0)	19 (50.0)	0.0049	0.0019	0.0038

^a^ Cachexia is defined as greater than 8% body weight loss, muscle radiation attenuation below the BMI‐dependent cutoff, and L3 skeletal muscle index below the sex‐ and BMI‐dependent cutoffs described by Martin et al in 2008; these individuals are a subset of subjects that lost more than 5% body weight.

^b^ Two‐tailed *t*‐test (continuous) or the Fisher's exact test (categorical) result of less than 8% body weight loss compared to control individuals.

^c^ Two‐tailed *t*‐test (continuous) or the Fisher's exact test (categorical) result of more than 8% body weight loss compared to control individuals.

^d^ Two‐tailed *t*‐test (continuous) or the Fisher's exact test (categorical) result of cachexia compared to control individuals.

**FIGURE 1:**
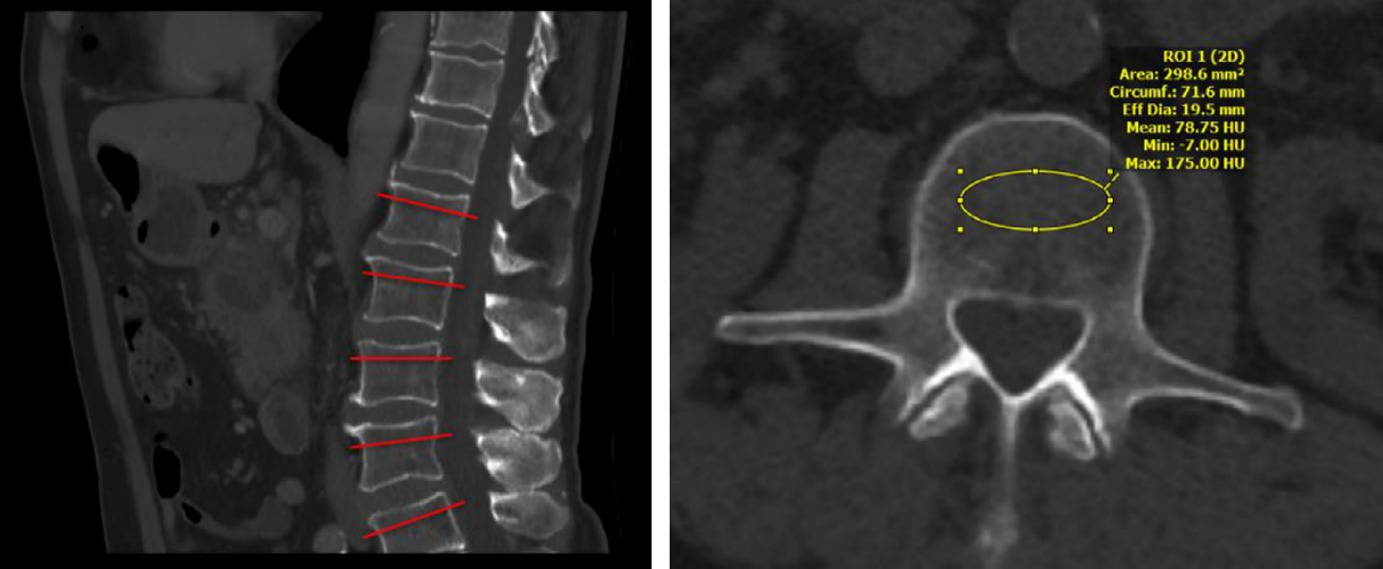
Determination of lumbar vertebral radiodensity. Osteopenia is assessed through routine CT scans using adjusted axial scans that capture the cross‐section of each lumbar (L1–L5) vertebra. Adjusting the scan image accounts for normal shape of the spine and patient position on the scanner bed. Lumbar vertebral radiodensity (LVR and HU) is recorded as the average of the radiation attenuation of the five vertebrae in a region of interest in the cancellous bone of the vertebral body

Bone density was approximated from the lumbar vertebral radiodensity (LVR and HU) using validated methods previously described.^26^, ^27^ In brief, five individual measurements were taken corresponding to the attenuation of a region of interest located in the body of each lumbar (L1–L5) vertebra (Figure 1). The scans were manually adjusted to accommodate for the individual's position on the scanner bed such that the cross‐section lay parallel to the superior aspect of the vertebral body and perpendicular to the lateral margins of the bone. LVR was the calculated as the mean value for the five images selected. Individuals were classified as osteopenic if the LVR was less than 145 HU.^26^ Evidence of cortical bone remodeling or spinal surgery precluded images from the analysis. In such cases, an average LVR was taken only from cross‐sectional images of healthy vertebrae.

### 2.2 Statistical analysis

All analyses were performed using SPSS version 22.0 (IBM Corp., Armonk, NY) and GraphPad Prism version 5.02 (GraphPad Software, San Diego, CA). Continuous variables were analyzed using independent sample *t*‐tests to compare means of two groups. One‐way analysis of variance tests were used to compare multiple means. Correlations were assessed assuming Gaussian distribution and with Pearson's coefficient of correlation. Cox–Mantel tests were used with generated Kaplan–Meier survival curves to determine the median survival. Cox proportional hazard ratios were calculated using univariate analysis for the long‐term survival using categorical values, such as presence or lack of osteopenia, or median values as a cutoff for continuous data. All mortality measures were specific to PDAC. Sensitivity and specificity for LVR and various cachexia measures were calculated to predict the survival at various time points postoperatively. Receiver operating characteristic (ROC) curves were plotted using sensitivity and 1–specificity; the area under the curve (AUC) was calculated. A *p* value less than 0.05 was considered significant for all statistical tests.

## 3 RESULTS

Patient characteristics and clinical variables are summarized in Tables 1 and 2. All laboratory values were measured at diagnosis. Mean (±SD) age for patients with PDAC was 69.0 (±9.61) years. A majority of patients were male (57.2%) and Caucasian (88.8%). No significant differences in age, sex, race, medication, or comorbidity status existed between control and PDAC patient groups. Furthermore, both cancer and control groups had similar prevalence of osteoporosis and vitamin D and calcium supplement use according to the clinical record. Tobacco smoking status, a risk factor for both PDAC and osteopenia, was also similar in both PDAC and control patient groups. No patients were taking bisphosphonates or other anti‐osteoporotic medications, such as denosumab or parathyroid hormone.

**TABLE 2:** Tumor pathologic variables

Tumor variable	Less than 5% body weight loss (*n* = 48)	More than 5% body weight loss (*n* = 104)	Cachexia (*n* = 38)	*p* value
Neoadjuvant therapy, *n* (%)	15 (31.3)	22 (21.2)	8 (21.1)	0.3617
T Stage				
1, *n* (%)	1 (2.08)	5 (4.81)	1 (2.63)	0.6586
2, *n* (%)	9 (18.8)	6 (5.77)	2 (5.26)	0.0225
3, *n* (%)	36 (75.0)	88 (84.6)	33 (86.8)	<0.0001
4, *n* (%)	2 (4.17)	5 (4.81)	2 (5.26)	0.9709
Node positive disease, *n* (%)	34 (70.8)	78 (75.0)	29 (76.3)	0.8155
Positive lymph node ratio	0.143 (0.171)	0.152 (0.158)	0.171 (0.185)	0.7335
Tumor histology				
Well‐differentiated, *n* (%)	5 (10.4)	9 (8.65)	3 (7.90)	0.9094
Moderately differentiated, *n* (%)	17 (35.4)	51 (49.0)	23 (60.5)	0.0646
Poorly differentiated, *n* (%)	24 (50.0)	39 (37.5)	9 (23.7)	0.0438
Undifferentiated, *n* (%)	2 (4.17)	4 (3.85)	3 (7.90)	0.5894
Positive resection margin, *n* (%)	13 (27.1)	26 (25.0)	8 (21.1)	0.8094
Cancer antigen 19‐9 (U/mL)	537 (1590)	413 (597)	486 (639)	0.7716

Weight loss was common in patients with PDAC and of the 152 PDAC patients included, 104 patients (68.4%) lost greater than 5% of their body weight in the 6 months preceding diagnosis, which is a criteria set forth by a panel of experts for a diagnosis of cachexia.^8^ However, a more recent classification demonstrated that cachectic cancer patients with involuntary body weight loss and two additional criteria—muscle depletion and low muscle attenuation, measured from CT images using tissue segmentation software—have significantly shorter survival irrespective of body weight.^29^ Using these more stringent criteria, 38 (25.0%) patients were classified as cachectic. The mean age of patients in this cachectic PDAC subgroup was 70.0 (±7.01) years and greater than control individuals (64.2 ± 12.6 years, *p* = 0.0297), but other demographic measures were similar across controls and the other PDAC groups.

### 3.1 Biochemical markers of cachexia

Hemoglobin, albumin, and platelet count were used as blood‐based markers of cachexia. PDAC patients with more than 5% body mass loss had a lower mean hemoglobin (12.4 ± 1.77 g/dl) level than control individuals (13.3 ± 1.56 g/dl; *p* = 0.0386). Mean hemoglobin was likewise lower in the subgroup with definitive cachexia (12.1 ± 1.69 g/dl, *p* = 0.0120) than control individuals. When considered categorically, 21.1% of control individuals and 50.0% of PDAC subjects were classified as anemic. There was a significant difference between control individuals and subjects with greater than 5% body weight loss (50.0%, *p* = 0.0242) and cachexia (52.6%, *p* = 0.0267). All patients with PDAC had higher mean corpuscular volume (MCV, 93.0 ± 5.63 fl) compared to control individuals (89.6 ± 3.27 fl, *p* = 0.0122); MCV was particularly elevated in PDAC patients groups with weight loss (93.2 ± 5.10 fl, *p* = 0.0039) and cachexia (92.8 ± 5.73 fl, *p* = 0.0310). Thus, and in spite of the elevated MCV, most PDAC patients displayed signs of normocytic anemia, typical of chronic disease.

Mean albumin level and platelet count were likewise altered in most PDAC subjects compared to controls. Subjects with less than 5% body weight loss (3.78 ± 0.555 g/dl, *p* = 0.0364) and cachexia (3.79 ± 0.459 g/dl, *p* = 0.0325) had lower serum albumin than controls (4.11 ± 0.597 g/dl). Collectively, PDAC subjects had higher mean platelet counts than control individuals (260 ± 101 vs. 202 ± 51.6, *p* = 0.0142). This remained consistent across all PDAC groups: less than 5% body weight loss (259 ± 95.8, *p* = 0.0172), more than 5% body weight loss (261 ± 104, *p* = 0.0167), and cachexia (253 ± 89.0, *p* = 0.0243).

All other recorded lab values and electrolytes (creatinine, sodium, and potassium) were similar between PDAC subjects and control individuals. As expected, aspartate aminotransferase (AST) tended to be elevated PDAC subjects (64.4 ± 85.3 U/L) with tumors leading to biliary obstruction compared to controls (37.8 ± 46.1 U/L) but did not achieve significance. However, compared to the reference range, control individuals also displayed a degree of hypertransaminasemia in blood assays. Serum calcium concentration also tended to be higher in most PDAC subjects (9.31 ± 0.707 mmol/L) than controls (9.07 ± 0.825 mmol/L) as would be expected during active and rapid bone resorption.

### 3.2 Body composition

Body mass index (BMI) was lower in PDAC subjects (26.6 ± 5.00 kg/m^2^, *p* = 0.0254) than controls (29.4 ± 4.69 kg/m^2^). BMI was most significantly decreased in patients with cachexia (25.7 ± 4.99 kg/m^2^, *p* = 0.0084). The average subject in this cohort lost 17.1% body weight. Together with low BMI and body weight loss, changes to lean body mass and adiposity are prognostic for poor outcomes in cancer.^29^ SMI at the L3 vertebral level was lower in all subjects with PDAC, significantly so in the cohort with cachexia (38.7 ± 5.99 cm^2^/m^2^), *p* < 0.0001 compared to controls (46.1 ± 5.77 cm^2^/m^2^). Other anthropometric measures of cachexia, notably MRA, were significantly altered between controls and PDAC subjects. Control individuals had a mean MRA of 34.5 ± 9.61 HU compared to 25.9 ± 5.95 HU in subjects with cachexia (*p* < 0.0001). When a prognostic MRA cutoff was used, both subjects with cachexia (100%, *p* < 0.0001) and those with more than 5% body weight loss (76.0%, *p* = 0.0239) had an increased prevalence of myosteatosis compared to control individuals (47.4%). There were no significant alterations to inter‐ or intra‐muscular adipose content in PDAC subjects compared to controls, nor sufficient radiologic evidence to support adipose wasting.

### 3.3 Signs of bone loss

**FIGURE 2:**
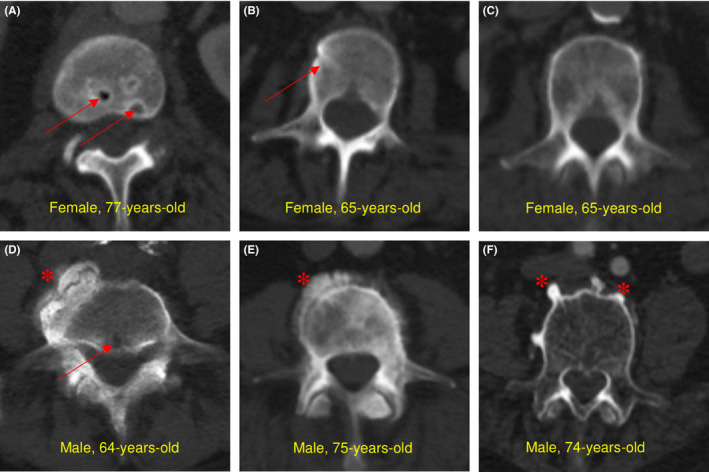
Bone degeneration in subjects. Several subjects (panels A‐F) had frank signs of bone degeneration when respective CT scans were analyzed. Common hallmarks of age were prevalent in both control and cancer groups, notably osteophytic changes at the periphery of the vertebral body. Osteophytes are present in all male examples and indicated by red asterisks. Other changes seemed to associate more directly with PDAC such as signs of remodeling and lytic lesions and are indicated by red arrows. Several examples visibly displayed attenuation changes to cancellous bone density. The bottom, right panel (F) displays markedly low density while the upper left (A), middle (B) and upper right (C) panels show increased attenuation. These latter examples also display signs of compression in the vertebral spine when viewed in the sagittal plane.

Upon accessing CT scans from the clinical record, several examples of bone loss were apparent in patients with PDAC. Importantly, there was no evidence of metastatic PDAC dissemination to bone. Indeed, metastatic disease precludes tumor removal by surgery and all patients studied underwent surgery with curative intent. Figure 2 documents examples of bone degeneration. Scans often displayed visibly low radiodensity in the cancellous bone of the vertebrae. Markedly low bone mineral density, observed radiologically, was combined with the presence of noteworthy lytic lesions, such as those observed in Figure 2A. As low bone mineral density is a predictor of fracture, we also assessed scans for evidence pathologic remodeling. Gross fractures were not apparent, but several patients had evidence of cortical bone remodeling and possible evidence of compression in the lumbar spinal column (Figure 2B,C,E). Compared to control individuals, those with PDAC were more likely to display signs of bone degeneration (49.3% vs. 10.5%, *p* = 0.0012). Notably, the L5 vertebra had the highest radiation attenuation (151 ± 47.7 HU, *p* = 0.0174) compared to L1 (138 ± 46.4 HU), L2 (137 ± 46.6), L3 (137 ± 45.6), and L4 (146 ± 48.1). In addition to these changes, numerous patients had osteophytes projecting from the vertebral body.

### 3.4 Quantified bone density

All PDAC groups demonstrated lower LVR than non‐cancer controls. The average LVR for control individuals was 166 ± 44.0 HU compared to 141 ± 36.6 HU (*p* = 0.0197) in those with less than 5% body weight loss, 141 ± 46.2 HU (*p* = 0.0330) in those with more than 5% body weight loss, and 129 ± 40.1 (*p* = 0.0025) in those with cachexia. Using pre‐established LVR cutoffs, we retrospectively diagnosed 61.2% of all PDAC patients with osteopenia. In total, 71.7% of patients with established cachexia were osteopenic compared to 36.8% of control individuals (*p* = 0.0214). While osteopenia is more common in otherwise healthy females, we did not identify statistically significant differences in bone density between males (138 ± 42.6 HU) and females (130 ± 36.1 HU) with PDAC, though values were numerically lower in females. Patients that received neoadjuvant chemotherapy (131 ± 6.82 HU) generally had lower LVR than those treated with upfront surgery (144 ± 4.16 HU), but the observed difference was not statistically significant (*p* = 0.1090).

**FIGURE 3:**
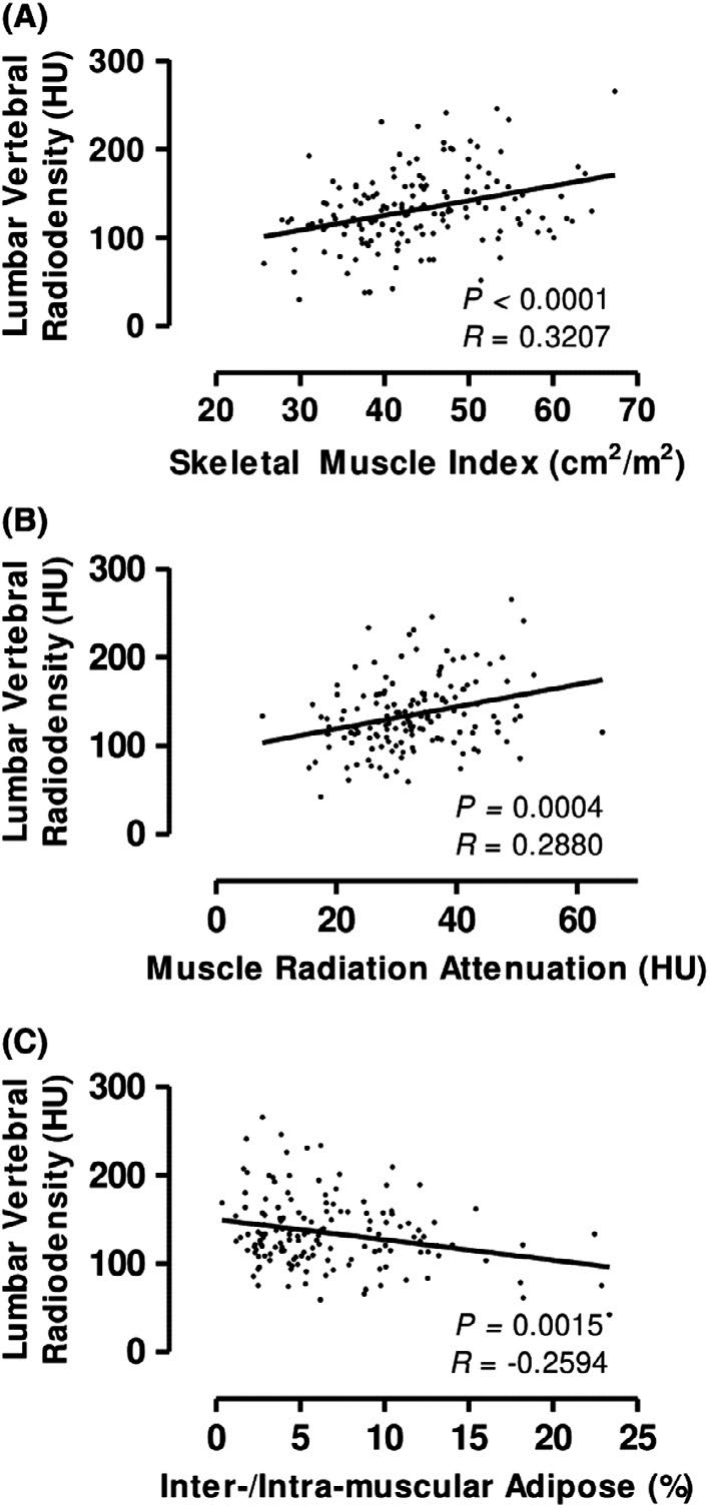
Correlations between lumbar vertebral radiodensity and cachexia measures. Anthropometric and blood‐based measures of cachexia correlate with LVR in subjects with PDAC. Greater LVR correlates positively with SMI (A) and MRA (B). Likewise, lower LVR is correlated with increased inter‐/intra‐muscular adipose deposition (C)

After identifying lower LVR and the high prevalence of osteopenia in patients with PDAC, we sought to identify correlations between bone density and other anthropometric cachexia measures (Figure 3). LVR significantly correlated with SMI (*p* < 0.0001), MRA (*p* = 0.0004), and inter‐/intra‐muscular adipose content (*p* = 0.0015). Curiously, LVR positively correlated with platelet count (*p* = 0.0469). LVR did not correlate with any additional anthropometric measures, blood chemistries, or tumor variables.

### 3.5 Survivorship and outcomes

**FIGURE 4:**
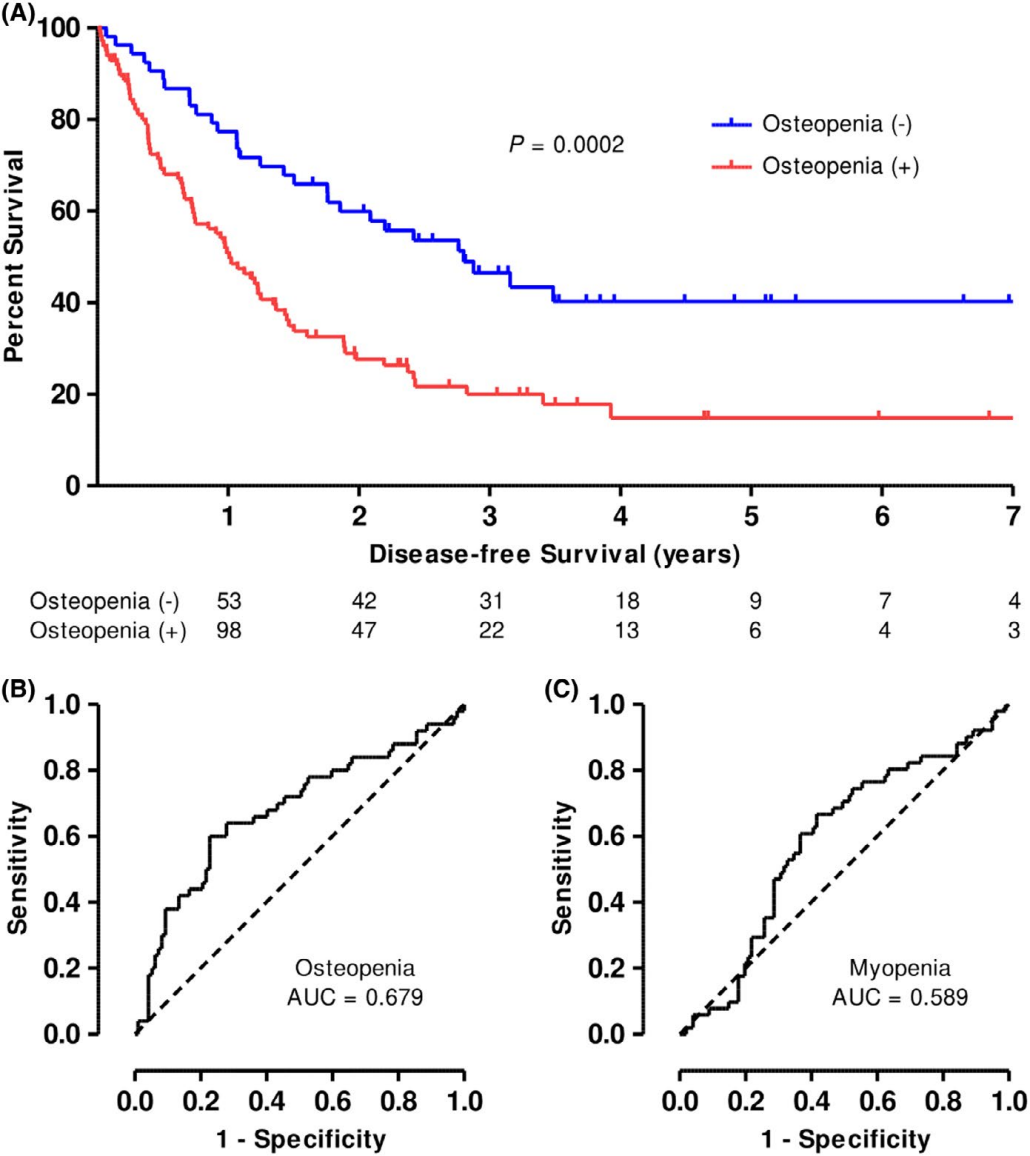
Osteopenia and long‐term, disease‐free survival. PDAC subjects with normal bone density have significantly greater disease‐free survival than their counterparts with radiologic osteopenia (A). Median survival for subjects with normal bone density was 2.77 years while those with bone wasting lived a median of 1.01 years. Receiver operating characteristic curves demonstrate that osteopenia (B) is a more sensitive and specific test of 2‐year survival than myopenia (C)

Median disease‐free survival for all patients with PDAC was 1.21 years. In total, 58.3% of patients were alive at 1 year, and 36.4% were alive at 2 years. When patients were categorized by osteopenia, key survival differences were noted through the Kaplan–Meier curves (Figure 4). Despite all patients undergoing potentially curative surgery, osteopenic patients had significantly reduced median survival (1.01 years) compared to those without osteopenia (2.80, *p* = 0.0002). Furthermore, osteopenia is the single most powerful predictor of survival at 1 and 2 years postoperatively with Cox proportional hazard ratios of 2.07 (CI: 1.22–3.52, *p* < 0.05) and 1.80 (CI: 1.17–2.77, *p* = 0.0153), respectively (Table 3). No other variables were significant in predicting disease‐specific mortality at 2 years, though low MRA also tended to predict early recurrence (HR: 1.47, CI: 0.972–2.23, *p* = 0.0715). Furthermore, preoperative osteopenia predicts 2‐year mortality with 76% sensitivity and 58% specificity. Receiver operating characteristic curves were plotted of LVR‐ and SMI‐derived categorical assignments of osteopenia and myopenia, respectively, as a test of 2‐year mortality. The area under the curve for osteopenia was 0.679 compared to 0.589 for myopenia. Osteopenia did not predict peri‐operative outcomes in our cohort of patients.

**TABLE 3:** Death hazard ratios at 2 years

Variable	Hazard ratio	95% confidence interval		*p* value
Osteopenia	1.80	1.17	2.77	0.0153
Low muscle radiation attenuation^a^	1.47	0.972	2.23	0.0715
Poor tumor differentiation	1.32	0.867	2.02	0.1872
High positive lymph node ratio^a^	1.26	0.832	1.91	0.2762
Advanced age^a^	1.24	0.818	1.88	0.3116
Large tumor size^a^	1.23	0.809	1.86	0.3401
Myopenia	1.20	0.781	1.84	0.4196
Positive operative margin	1.19	0.741	1.92	0.4501
Anemia	0.879	0.580	1.33	0.5437
No neoadjuvant therapy	0.903	0.559	1.46	0.6684
Hypoalbuminemia^a^	1.02	0.687	1.51	0.9238
High platelet count^a^	1.02	0.672	1.54	0.9312

^a^ Continuous data cutoffs are defined by their relation relative to the median value.

## 4 DISCUSSION

Cachexia is common in PDAC. As our present study indicates, a majority of patients experience weight loss in the months preceding diagnosis. Key changes to body composition are common, such as reductions in skeletal muscle mass and quality. Our study further indicates that patients with resectable PDAC have markedly reduced bone density compared to age‐matched individuals. Furthermore, alterations in bone density correlate strongly with commonly described anthropometric measures of cachexia, including skeletal muscle mass (SMI), muscle quality (MRA), and inter‐/intra‐muscular adipose percent infiltration. We therefore describe osteopenia as a novel aspect of systemic wasting in cancer that occurs synchronously with reductions in the health of skeletal muscle. In the current study, low bone density also served as the single strongest predictor of long‐term, disease‐free survival in patients that underwent curative surgery for PDAC. Osteopenic patients were 107% more likely to die within 1 year and 80% more likely to die within 2 years of surgery than those with normal bone density. Our methods of measuring bone density serve as a prognostic test of 2‐year mortality in patients following surgery for PDAC.

To our knowledge, there have been no previous reports of low bone density or bone atrophy in a clinical population of patients with hepatopancreaticobiliary or gastrointestinal cancer. Various preclinical models of cancer cachexia, however, have identified bone depletion as a phenomenon of the wasting phenotype. Key murine models of pancreatic, colorectal, and lung cancers display decreases in global bone mineral density, bone mineral content, femoral trabecular bone volume fraction, and trabecular number.^21^, ^23^, ^30^ When interrogated through three‐point bending tests, animal models of familial adenomatous polyposis (APC^−/+^) exhibit functional deficits in bone strength.^21^ Furthermore, when various cytokines that contribute to cancer cachexia—such as TGF‐β, IL‐6, and tumor necrosis factor alpha (TNF‐alpha)—are inhibited, bone mineral density loss is attenuated, most likely by preventing full differentiation of osteoclasts through the RANK–RANKL pathway.^30^, ^31^, ^32^ In brief, the scientific consensus states that inflammation of any cause is likely to cause substantial bone density loss.^33^ As cancer and cachexia are intrinsically pathologies of inflammation, it is reasonable to associate the diverse clinical manifestations of wasting and osteopenia through the various causative inflammatory cytokines. A chronically elevated inflammatory state is supported by the high prevalence of anemia of chronic disease and thrombocytosis in our patient population.

Human studies of body composition often focus on correlative analyses that might predict cancer outcomes. Most often, these studies rely upon multiple measurements across time to determine the systemic response to various anticancer treatments. Bone density has been studied longitudinally in patients receiving neoadjuvant chemotherapy, radiation, and definitive surgery for PDAC.^19^ Continuous loss of bone postoperatively predicted earlier tumor recurrence when compared to patients with constant bone mineral density. In other words, circulating tumor stem cells and their interaction with host cells may facilitate the release of inflammatory factors that promote osteoclast differentiation. Indeed, IL‐6 and interleukin‐8 (IL‐8) promote PDAC stem cell colony formation and metastasis.^34^, ^35^ The clinical reality of cancer care and bone density change rely upon a better understanding of the pathogenesis of osteopenia in cancer.

Our present study is not without limitation. Reported observations were made retrospectively in a selected group of surgical patients with PDAC from one geographical region. Furthermore, we selected patients with benign gallbladder disease to serve as our control population because in our hepatopancreaticobiliary surgical practice, the most common benign surgical indication is for gallbladder disease. Therefore, clinical CT scans are available from this non‐cancer control population for this retrospective study, but not available from healthy controls, and we have used similar controls in other publications by our group.^36^ We do, however, acknowledge that in our comparisons between PDAC and controls, our findings could be impacted by the selection of gallbladder disease patients as our non‐cancer controls. Thus, prospective studies should be considered in the future, such that healthy controls can be included.

Though we have established impaired bone biology as a paraneoplastic element of cancer, we did not attempt to ascertain the mechanisms underpinning this clinical scenario. How do tumor, muscle, and bone interact? Might treating cancer patients with the various FDA‐approved osteoporosis medications, such as denosumab, influence survivability? We have performed correlative analyses between established markers of cachexia and our novel assessment of bone density, but our observations have been made at only one—the preoperative—time point. Thus, we are unable to place the onset of cachexia in the continuum that is the pathway from pre‐cachexia to refractory, end‐stage wasting. In future clinical trials, we will interrogate blood‐based biospecimens and tumor and muscle biopsies to determine circulating and tumor‐derived factors that may be related to systemic wasting, such as the various inflammatory cytokines as well as the markers of bone formation and resorption.^10^, ^37^ Specific endocrine factor levels in the bone–muscle axis, such as androgens, cortisol, osteocalcin, and IGF‐1, will also be determined and studied with clinical imaging and quantification of bone density.^16^, ^38^, ^39^, ^40^ Such studies could likewise consider any sex‐based differences in PDAC‐induced osteopenia, including whether levels of sex hormones and their receptors relate to such outcomes. In addition, further assessment of bone density should be performed in other cancers that commonly cause cachexia, such as esophageal and lung cancers. Further studies should likewise assess bone mineral density in metastatic gastrointestinal cancers and the role of anticancer treatments. Relatively common confounding variables contributing to reduced bone mineral density such as vitamin D deficiency or steatorrhea (leading to a reduction in ability to absorb fat‐soluble vitamins) need to be prospectively isolated as contributing causes. Reductions in muscle mass related to sedentary activity levels could also be contributing but would likely require months or years to contribute to osteopenia. Such trials should also include routine dual‐energy x‐ray absorptiometry (DXA) scans to evaluate their prognostic significance. Beyond the clinical realm, we also propose that bone density changes should be examined specifically in the sundry models of cancer cachexia and tumor biology to ascertain the biologic mechanisms of paraneoplastic osteopenia.

In summary, we report that osteopenia is a novel facet of systemic wasting in patients with resectable PDAC. Multiple anthropometric changes can be measured in the months preceding diagnosis. These measures most often consider skeletal muscle mass and quality. In our study, we demonstrate that bone density correlates with noninvasive measures of skeletal muscle pathology. As an independent element of cachexia, impaired bone density portents poor surgical and oncologic outcomes. Patients with osteopenia have reduced survival. Indeed, the presence of osteopenia serves as the single strongest predictor of mortality in our clinical population. If validated, osteopenia should be considered a stratification factor in clinical trials testing the perioperative management of PDAC. Thus, from the findings reported herein, we define osteopenia as a pertinent clinical element of cancer cachexia.

## ETHICS STATEMENT

All research subjects were recruited and provided written informed consent as per Institutional Review Board guidelines at the University of Florida in Gainesville, FL.

## CONFLICT OF INTEREST

The authors report no conflict of interest. This was not an invited submission.

## AUTHOR CONTRIBUTION

MEC, PWU, SMJ, JGT, and ARJ conceptualized and designed the study. MEC, PWU, IEW, JGT, and ARJ curated the data. MEC, IEW, JGT, and ARJ performed formal analyses. MEC and ARJ wrote the manuscript. MEC, PWU, TJG, JFY, and ARJ reviewed and edited the manuscript. All authors approved the final manuscript.

## Data Availability

The data that support the findings of this study are available upon request from the corresponding author. The data are not publicly available due to privacy or ethical restrictions.

## References

[1] American Cancer Society . Cancer Facts & Figures 2020. American Cancer Society; 2020.

[2] Rahib L , Smith BD , Aizenberg R , Rosenzweig AB , Fleshman JM , Matrisian LM . Projecting cancer incidence and deaths to 2030: the unexpected burden of thyroid, liver, and pancreas cancers in the United States. Cancer Res. 2014;74(11):2913‐2921. 10.1158/0008-5472.CAN-14-0155 24840647

[3] Hendifar AE , Chang JI , Huang BZ , Tuli R , Wu BU . Cachexia, and not obesity, prior to pancreatic cancer diagnosis worsens survival and is negated by chemotherapy. J Gastrointest Oncol. 2018;9(1):17–23. 10.21037/jgo.2017.11.10 29564167 PMC5848037

[4] Kahl C , Krahl R , Becker C , et al. Long‐term follow‐up of the AML97 study for patients aged 60 years and above with acute myeloid leukaemia: a study of the East German Haematology and Oncology Study Group (OSHO). J Cancer Res Clin Oncol. 2016;142:305–315. 10.1007/s00432-015-2045-8 26407768 PMC11819363

[5] Baracos VE , Martin L , Korc M , Guttridge DC , Fearon KCH . Cancer‐associated cachexia. Nat Rev Dis Primers. 2018;4(1):17105. 10.1038/nrdp.2017.105 29345251

[6] Bachmann J , Heiligensetzer M , Krakowski‐Roosen H , Buchler MW , Friess H , Martignoni ME . Cachexia worsens prognosis in patients with resectable pancreatic cancer. J Gastrointest Surg. 2008;12(7):1193‐1201. 10.1007/s11605-008-0505-z 18347879

[7] Delitto D , Judge SM , George TJ , et al. A clinically applicable muscular index predicts long‐term survival in resectable pancreatic cancer. Surgery. 2017;161(4):930‐938. 10.1016/j.surg.2016.09.038 27932030

[8] Fearon K , Strasser F , Anker SD , et al. Definition and classification of cancer cachexia: an international consensus. Lancet Oncol. 2011;12(5):489‐495. 10.1016/S1470-2045(10)70218-7 21296615

[9] Evans WJ , Morley JE , Argiles J , et al. Cachexia: a new definition. Clin Nutr. 2008;27(6):793‐799. 10.1016/j.clnu.2008.06.013 18718696

[10] Tisdale MJ . Mechanisms of cancer cachexia. Physiol Rev. 2009;89(2):381‐410. 10.1152/physrev.00016.2008 19342610

[11] Yakabe M , Hosoi T , Akishita M , Ogawa S . Updated concept of sarcopenia based on muscle‐bone relationship. J Bone Miner Metab. 2020;38(1):7‐13. 10.1007/s00774-019-01048-2 31583540

[12] Curtis E , Litwic A , Cooper C , Dennison E . Determinants of muscle and bone aging. J Cell Physiol. 2015;230(11):2618‐2625. 10.1002/jcp.25001 25820482 PMC4530476

[13] Laurent MR , Dubois V , Claessens F , et al. Muscle‐bone interactions: from experimental models to the clinic? A critical update. Mol Cell Endocrinol. 2016;432:14‐36. 10.1016/j.mce.2015.10.017 26506009

[14] Tagliaferri C , Wittrant Y , Davicco M‐J , Walrand S , Coxam V . Muscle and bone, two interconnected tissues. Ageing Res Rev. 2015;21:55‐70. 10.1016/j.arr.2015.03.002 25804855

[15] Argiles JM , Stemmler B , Lopez‐Soriano FJ , Busquets S . Inter‐tissue communication in cancer cachexia. Nat Rev Endocrinol. 2018;15(1):9‐20. 10.1038/s41574-018-0123-0 30464312

[16] Karsenty G , Olson EN . Bone and muscle endocrine functions: unexpected paradigms of inter‐organ communication. Cell. 2016;164(6):1248‐1256. 10.1016/j.cell.2016.02.043 26967290 PMC4797632

[17] Petzel MQB , Hoffman L . Nutritional implications for long‐term survivors of pancreatic cancer surgery. Nutr Clin Pract. 2017;32(5):588–598. 10.1177/0884533617722929 29927530

[18] Zhang Q , Sun X , Yang J , et al. ZIP4 silencing improves bone loss in pancreatic cancer. Oncotarget. 2015;6(28):26041‐26051. 10.18632/oncotarget.4667 26305676 PMC4694884

[19] Yamada D , Eguchi H , Iwagami Y , et al. Patients treated with preoperative chemoradiation for pancreatic ductal adenocarcinoma have impaired bone density, a predictor of distant metastasis. Ann Surg Oncol. 2017;24(12):3715‐3724. 10.1245/s10434-017-6040-y 28849575

[20] Waning DL , Mohammad KS , Reiken S , et al. Excess TGF‐ β mediates muscle weakness associated with bone metastases in mice. Nat Med. 2015;21(11):1262‐1271. 10.1038/nm.3961 26457758 PMC4636436

[21] Bonetto A , Kays JK , Parker VA , et al. Differential bone loss in mouse models of colon cancer cachexia. Front Physiol. 2017;7. 10.3389/fphys.2016.00679 PMC5225588 28123369

[22] Pin F , Barreto R , Kitase Y , et al. Growth of ovarian cancer xenografts causes loss of muscle and bone mass: a new model for the study of cancer cachexia. J Cachexia Sarcopenia Muscle. 2018;9(4):685‐700. 10.1002/jcsm.12311 30009406 PMC6104117

[23] Choi E , Carruthers K , Zhang L , et al. Concurrent muscle and bone deterioration in a murine model of cancer cachexia. Physiol Rep. 2013;1(6):e00144. 10.1002/phy2.144 24400146 PMC3871459

[24] Shen W , Punyanitya M , Wang Z , et al. Visceral adipose tissue: relations between single‐slice areas and total volume. Am J Clin Nutr. 2004;80(2):271‐278. 10.1093/ajcn/80.2.271 15277145 PMC2040041

[25] Aubrey J , Esfandiari N , Baracos VE , et al. Measurement of skeletal muscle radiation attenuation and basis of its biological variation. Acta Physiol (Oxf). 2014;210(3):489‐497. 10.1111/apha.12224 24393306 PMC4309522

[26] Pickhardt PJ , Lee LJ , Munoz del Rio A , et al. Simultaneous screening for osteoporosis at CT colonography: bone mineral density assessment using MDCT attenuation techniques compared with DXA reference standard. J Bone Miner Res. 2011;26(9):2194–2203. 10.1002/jbmr.428 21590738 PMC3304444

[27] Weishaupt D , Schweitzer ME , DiCuccio MN , Whitley PE . Relationships of cervical, thoracic, and lumbar bone mineral density by quantitative CT. J Comput Assist Tomogr. 2001;25(1):146‐150. 10.1097/00004728-200101000-00027 11176311

[28] Dindo D , Demartines N , Clavien PA . Classification of surgical complications: a new proposal with evaluation in a cohort of 6336 patients and results of a survey. Ann Surg. 2004;240(2):205‐213. 10.1097/01.sla.0000133083.54934.ae 15273542 PMC1360123

[29] Martin L , Senesse P , Gioulbasanis I , et al. Diagnostic criteria for the classification of cancer‐associated weight loss. J Clin Oncol. 2015;33(1):90‐99. 10.1200/JCO.2014.56.1894 25422490

[30] Greco SH , Tomkotter L , Vahle A‐K , et al. TGF‐β blockade reduces mortality and metabolic changes in a validated murine model of pancreatic cancer cachexia. PLoS One. 2015;10(7):e0132786. 10.1371/journal.pone.0132786 26172047 PMC4501823

[31] Schett G . Effects of inflammatory and anti‐inflammatory cytokines on the bone. Eur J Clin Invest. 2011;41(12):1361‐1366. 10.1111/j.1365-2362.2011.02545.x 21615394

[32] Udagawa N , Takahashi N , Katagiri T , et al. Interleukin (IL)‐6 induction of osteoclast differentiation depends on IL‐6 receptors expressed on osteoblastic cells but not on osteoclast progenitors. J Exp Med. 1995;182(5):1461‐1468. 10.1084/jem.182.5.1461 7595216 PMC2192181

[33] Hardy R , Cooper MS . Bone loss in inflammatory disorders. J Endocrinol. 2009;201(3):309‐320. 10.1677/JOE-08-0568 19443863

[34] Fu S , Lin J . Blocking interleukin‐6 and interleukin‐8 signaling inhibits cell viability, colony‐forming activity, and cell migration in human triple‐negative breast cancer and pancreatic cancer cells. Anticancer Res. 2018;38(11):6271‐6279. 10.21873/anticanres.12983 30396947

[35] Lenk L , Pein M , Will O , et al. The hepatic microenvironment essentially determines tumor cell dormancy and metastatic outgrowth of pancreatic ductal adenocarcinoma. Oncoimmunology. 2017;7(1):e1368603. 10.1080/2162402X.2017.1368603 29296518 PMC5739558

[36] Judge SM , Nosacka RL , Delitto D , et al. Skeletal muscle fibrosis in pancreatic cancer patients with respect to survival. JNCI Cancer Spectrum. 2018;2(3):pky043. 10.1093/jncics/pky043 30637373 PMC6322478

[37] Callaway CS , Delitto AE , Patel R , et al. IL‐8 released from human pancreatic cancer and tumor‐associated stromal cells signals through a CXCR2‐ERK1/2 axis to induce muscle atrophy. Cancers (Basel). 2019;11(12):1863. 10.3390/cancers11121863 PMC6966692 31769424

[38] Carson JA , Manolagas SC . Effects of sex steroids on bones and muscles: similarities, parallels, and putative interactions in health and disease. Bone. 2015;80:67‐78. 10.1016/j.bone.2015.04.015 26453497 PMC4600533

[39] Misra M , Klibanski A . Endocrine consequences of anorexia nervosa. Lancet Diabetes Endocrinol. 2014;2(7):581‐592. 10.1016/S2213-8587(13)70180-3 24731664 PMC4133106

[40] Gomarasca M , Banfi G , Lombardi G . Myokines: the endocrine coupling of skeletal muscle and bone. Adv Clin Chem. 2020;94:155–218. 10.1016/bs.acc.2019.07.010 31952571

